# Arrhythmogenic properties of dismantling cadherin-mediated adhesion in murine hearts^☆^

**DOI:** 10.1016/S1674-8301(10)60041-3

**Published:** 2010-07

**Authors:** Hongjun Zhu, Hegui Wang, Xiwen Zhang, Xiaofeng Hou, Kejiang Cao, Jiangang Zou

**Affiliations:** Department of Cardiology, the First Affiliated Hospital of Nanjing Medical University, Nanjing 210029, Jiangsu Province, China

**Keywords:** adherence junction, N-cadherin, gap junction, ventricular tachyarrhythmia, recombinant mouse aminopeptidase N

## Abstract

**Objective:**

To evaluate the arrhythmogenic effects of dismantling cadherin-mediated adhesion by recombinant mouse aminopeptidase N (rmAPN) in murine hearts.

**Methods:**

rmAPN was incubated with cultured neonatal rat cardiomyocytes as well as being infused in adult mice. The cell-cell connections were immunolabelled and observed by laser confocal microscopy. Disruption of the N-terminal of N-cadherin (N-cad) was detected by western blot and quantitative immunofluorescence. The risk of inducible ventricular tachyarrhythmia was evaluated in mice by an electrophysiological study.

**Results:**

Disrupted cell-cell contact was observed in cultured neonatal rat cardiomyocytes in response to 30-40 ng/µL rmAPN. Loss of the N-terminal in N-cad and altered distribution of connexin 43 (Cx43) were observed in hearts from rmAPN-infused mice. In addition, a reduction of phosphorylated Cx43 was also detected concomitant with redistribution of Cx43. Electrophysiological studies of rmAPN-infused mice showed prolonged QRS duration and increased inducibility of ventricular tachycardias.

**Conclusion:**

Disruption of N-cad by rmAPN contributes to gap junction remodeling and may elicit arrhythmogenic effects. The disorder of adherent junctions by proteolytic enzymes may play an important role in arrhythmogenic mechanisms in correlated diseases.

## INTRODUCTION

Adherence junctions, gap junctions (GJs) and desmosomes must be properly organized in the intercalated disc (ID) to mediate normal mechanical and electrical coupling between the individual cardiomyocytes in the heart[1]. Adherence junctions are essential for the accumulation of connexin 43 (Cx43), which forms GJs relaying electrical coupling between cardiomyocytes[2]. Previous studies showed that abnormal mechanical joint proteins might cause impairment of adherence junctions and increase incidences of ventricular tachyarrhythmias[2],[3]. It was also demonstrated that proteolytic enzymes, including aminopeptidase and metalloproteases (MMPs) are elevated in infarcted heart tissue[4],[5]. A recent study showed that the proteolytic enzymes played an important role in ventricular tachyarrhythmias post myocardial infarction (MI)[6]. However, the effects of proteolytic enzymes on the cell-to-cell adhesion and intercellular electrical conduction have not been clarified.

N-cadherin (N-cad) is the most important transmembrane component building up adherence junctions in the ID. As one of the classical cadherins, N-cad is a single-pass transmembrane protein with five extracellular domains, one transmembrane domain and one cytoplasmic domain. The extracellular domains of N-cad are essential for homophilic calcium-inducible intercellular adhesion[7],[8]. When most of the extracellular domains of N-cad are deleted, adhesion function is compromised[9],[10]. The extracellular domains of N-cad may be degraded by various proteolytic enzymes, including MMPs[11], disintegrin and MMPs[12],[13]. Unlike N-cad, Cx43 is a four-transmembrane spanning protein. Both amino- and carboxyl-terminals of the Cx43 are in the cytoplasm[14]. Cx43 is not likely to be damaged by proteolytic enzymes which cleave the peptide from either of the two terminals.

In light of the importance of cell adhesion molecules in the maintenance of the electrical coupling in cardiomyocytes, we hypothesized that impairment of adherence junctions by proteolytic enzymes might influence the distribution of Cx43 and the function of GJs, resulting in a propensity for ventricular arrhythmias. In our study, the recombinant mouse aminopeptidase N (rmAPN), an enzyme preferentially releasing neutral amino acids from the N-terminal of oligopeptides, was used to cleave the extracellular N-terminal of N-cad in adult mice. The redistribution and dephosphorylation of Cx43, and increased incidences of inducible ventricular tachyarrhythmias were detected in rmAPN-treated mice.

## MATERIALS AND METHODS

### Animals

Neonatal Sprague-Dawley-Javanovas rats (1-2 d) and ICR male mice (6-8 w) were provided by the Animal Research Centre of Nanjing Medical University (Nanjing, China). All adult mice were housed at (23±2)°C and (70%±5%) humidity under a 12-h light/dark cycle. Mice were given a standard diet for at least 2 d as an acclimation period. The study was performed in accordance with the “Guide for the Care and Use of Laboratory Animals” (NIH Publication No. 85-23, National Academy Press, revised 1996). The experimental protocol was approved by the Animal Care and Use Committee of Nanjing Medical University.

### Antibodies and reagents

Primary antibodies used in the confocal immunoflu-orescence study included the following: rabbit polyclonal anti-Cx43 antibody (ab11370, Abcam Inc., USA), mouse monoclonal anti-Cx43 antibody (ab11369, Abcam Inc.), rabbit polyclonal anti-N-cad antibody (which reacts with epitopes on the N-terminal of N-cad, sc-7939, Santa Cruz Biotechnology, USA), and mouse monoclonal anti-pan cadherin antibody (which reacts with epitopes on the C-terminal of N-cad, ab6528, Abcam Inc.). Antibodies used in immunoblot analyses included the rabbit polyclonal anti-Cx43 antibody, the anti-N-cad antibodies mentioned above, a rabbit polyclonal anti-phosphorylated-Cx43 antibody (ab62252, Abcam Inc.) and a mouse monoclonal anti-GADPH antibody (sc-51906, Santa Cruz Biotechnology). Rhodamine-conjugated goat anti-rabbit and FITC-conjugated goat anti-mouse antibodies were gotten from Sigma-Aldrich, USA. HRP-conjugated goat anti-mouse and anti-rabbit antibodies were purchased from Santa Cruz Biotechnology.

Reagents used in this study were: rmAPN (residues 69-966, catalog# 2335-ZN, R&D Systems, USA); the penicillin/streptomycin (Amimed, Switzerland); 1-β-D arabinofuranosyl-cytosine was purchased from Sigma; fetal calf serum from Hyclone (Thermo Scientific, USA).

### Cell culture and interventions

Cardiac myocytes were isolated from the neonatal rats using enzymatic dissociation and were further purified by differential adhesion as described elsewhere[7]. The neonatal cardiomyocyte culture medium consisted of M199, 20% fetal calf serum, and 1% penicillin/streptomycin. To inhibit fibroblast growth, 1-β-D arabinofuranosyl-cytosine (10 µmol/L) was added. Cells were seeded in dishes at a density of (0.8-1)×10^5^ per square centimeter. Following 72 h of culture at 37°C in a humidified 5% CO_2_ incubator, activated rmAPN was added into the medium at the concentration of 10, 20, 30 and 40 ng/µL for 1 h. Cells were then fixed in 4% paraformaldehyde/PBS, and immunofluorescence labeling of both Cx43 and N-cad was carried out prior to laser confocal microscope inspection.

### Infusion of rmAPN and electrophysiological study *in vivo*

Mice were anesthetized with pentobarbital (70 mg/kg) and infused with rmAPN at concentration of 20 ng/µL *via* a jugular vein catheter attached to a peristaltic pump to maintain a constant flow rate of 2 µL/min. The control group mice were infused with saline. Electrocardiogram was monitored during the experimental period.

Electrophysiological studies were performed immediately after the completion of the 1 h infusion as previously described[15]. The programmed electric stimulation was administrated by RM6240 B/C physiological signal handling system 2.0 (Chengdu Instrument Corp., China), and the electronically stored traces were recorded and analyzed using the same instrument. Briefly, bipolar voltage pacing thresholds were measured with a 2-ms pulse width. The output amplitude was set at 200% of the stimulating threshold. Programmed electric stimulation (PES) was used to induce ventricular arrhythmias. PES consisted of a train of 8-beats with a basic cycle length of 100 ms followed by one extrastimulus (S1S2). The interval of the extrastimulus was shortened stepwise by 2 ms from 60 ms to 20 ms. If a ventricular arrhythmia was not induced, S1S2S3, S1S2S3S4 or burst pacing (from 1 to 35 Hz) was subsequently introduced. Ventricular arrhythmia included ventricular fibrillation (VF) and sustained ventricular tachycardia (SVT, that lasted more than 3 s). VF was defined as a polymorphic QRS complex with no clearly definable rate. VT was defined as discrete complexes with a clearly definable rate. The mice were sacrificed at the end of electrophysiological study. The hearts were collected for tissue immunofluorescence and immunoblot as described below.

### Confocal immunofluorescence microscopic inspection

The snap-frozen sections from 4 hearts of either group were double immunolabelled with antibodies of Cx43 and N-cad[16]. The specific immunofluorescent signals of Cx43 and N-cad at IDs were examined by a confocal immunofluorescence microscope (Zeiss LSM510 META, Germany) and quantified by Image-Pro Plus 6.0. Each tested area was digitized into a 1,024×1,024 matrix (1.05×10^6^ pixels/test area). Areas for analysis consisted of well-preserved, compact bundles of left ventricular myocytes in planes paralleled to the long axis of cells. Quantified analysis was limited to large, clearly identifiable intercalated disks. The signal area was quantified by counting the number of pixels that exhibited high signal intensity and was expressed as per unit disk length[16]. The colors of the signals (red or green) were distinguished as the location of the proteins been labelled, or the coexistent area of both antigens (yellow). The examiners were blinded to the group identity.

### Western blot analysis

The total content of N-cad and Cx43 were detected by immunoblot as described previously[17]. In brief, isolated hearts were snap frozen in liquid nitrogen, pulverized, and homogenized in hydrogencarbonate solution. Aliquots of protein were added to 4×sample buffer and resolved by sodium dodecyl sulfate-polyacrylamide (10%) gel electrophoresis and transferred (semi-dry) to nitrocellulose membranes. Membranes were blocked in 5% nonfat, dry milk in Tris-buffered saline containing 0.5‰ Tween 20 at room temperature for 2 h, and then incubated with rabbit anti-Cx43 (1:4,000), rabbit anti-p-Cx43 (1:2,000), mouse anti-GADPH (1:4,000), rabbit anti-N-cad (1:2,000) or the mouse anti-N-cad (1:2,000) antibodies overnight at 4°C. HRP-conjugated goat anti-mouse and anti-rabbit antibodies were used as secondary antibodies. GAPDH was used as the marker of protein loading. Density of individual bands were quantified as the ratio to GAPDH.

### Statistical analysis

All variables were expressed as mean±SD. Comparisons between the groups were analyzed using the Student's *t*-test. Comparison of the frequency of inducible ventricular arrhythmia between groups was performed using the Fisher exact test. Differences were considered significant when the *P*-value was less than 0.05.

## RESULTS

### rmAPN impairs cell-to-cell adhesion and alters the distribution of Cx43 *in vitro*

**Fig. 1:**
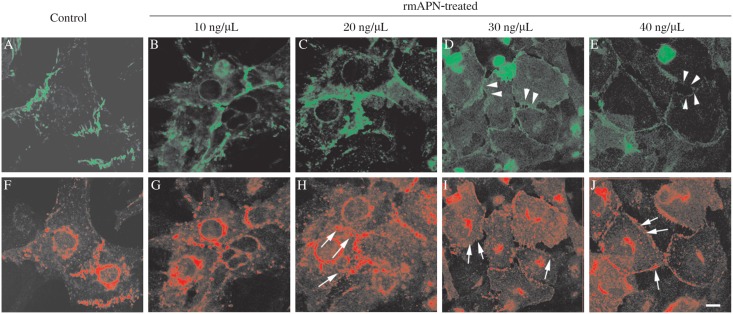
Influence of various concentrations of rmAPN on cell-cell adhesion and Cx43 distribution *in vitro*. Cultured neonatal rat cardiomyocytes were incubated with or without rmAPN and were double-immunostained with N-cad (green, A-E) and Cx43 (red, F-J). A and F: Normal cell-to-cell connections. N-cad and Cx43 signals concentrated in the connections between adjacent cells. B and G: Cells incubated with rmAPN (10 ng/µL). C and H: Cells incubated with rmAPN (20 ng/µL) display Cx43 signal pulling away from the cell-cell junctions and distributed into the cytoplasm (arrows). D and I: Cells incubated with 30 ng/µl rmAPN showing decreased N-cad signals, broken cell-cell connections (arrowheads), and diffused distribution of Cx43 (arrows) into the plasmalemma. E and J: Cells incubated with 40 ng/µL rmAPN. Scale bar = 10 µm, for all the panels.

To evaluate the effect of rmAPN on the cell-to-cell adhesion, cultured neonatal rat cardiomyocytes were incubated with different concentrations of rmAPN for 1 h. The cells were double-labelled with anti-Cx43 and anti-N-cad antibody and observed by laser confocal microscopy. The signals of Cx43 in the cytoplasm increased at the concentrations of 10 ng/µL and 20 ng/µL rmAPN without obviously abnormal adherence junctions. However, the broken cell-to-cell adhesions and strikingly diffused distribution of Cx43 on the plasmalemma were noted at the concentrations of 30 ng/µL and 40 ng/µL rmAPN (***Fig. 1***).

### rmAPN cleaves N-cad in the intercalated discs of murine cardiac tissue

**Fig. 2:**
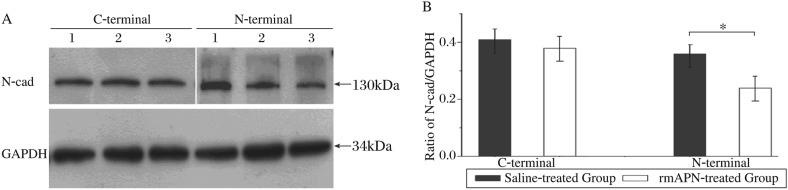
rmAPN involvement in dismantling N-cad. A: Representative immunoblot of cardiac tissues from N-cad of rmAPN- and saline-treated mice probed with anti-N-cad antibodies. Lane 1 was from saline-treated group; Lane 2 and 3 were different cases from rmAPN-treated group. B: Densitometric scanning results and quantitative analysis of the N-cad levels (*n* = 6, *P < 0.05).

To inspect the disrupted N-terminal of N-cad in the mice infused with rmAPN, antibodies which combine either the N-terminal or C-terminal of N-cad were used in western blot. Signals of N-cad were detected as the bands near 130 kDa despite the different primary antibodies (***Fig. 2A***). The N-cad protein level significantly decreased using anti-N-terminal antibody in rmAPN-treated mice compared to saline-treated mice (0.360±0.031 *vs* 0.240±0.049, *n* = 6, *P* < 0.01). However, the N-cad protein level was similar when examined with anti-C-terminal antibody in both groups (0.410±0.048 *vs* 0.380±0.065, *n* = 6, *P* > 0.05) (***Fig. 2B***). Therefore, rmAPN can disrupt the N-terminal of N-cad while keeping the C-terminal intact in murine heart tissue.

**Fig. 3:**
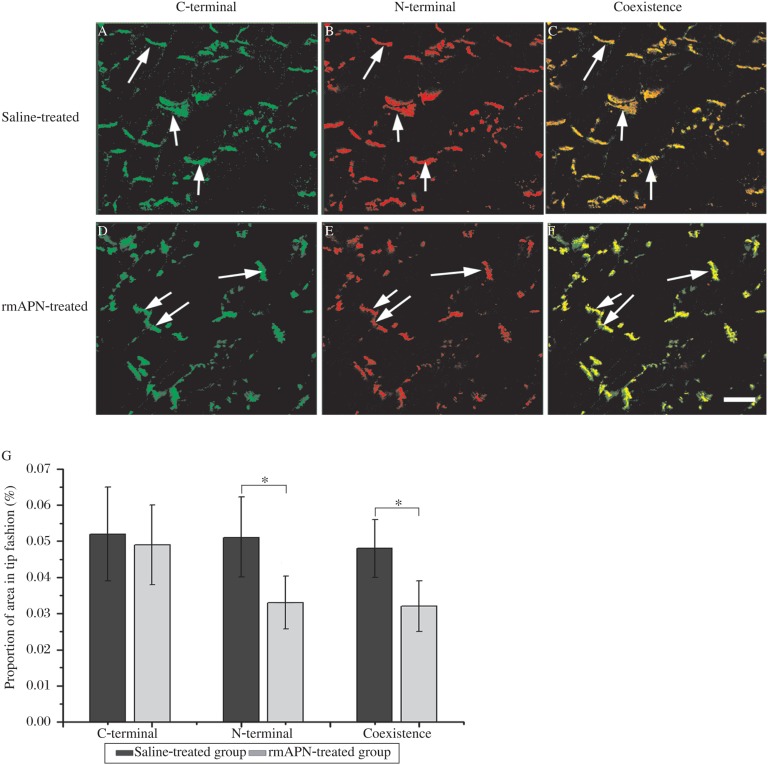
Quantitative immunofluorescence study on both terminals of N-cad. A-F: Immunofluorescence of C-terminal (green) and N-terminal (red) of N-cad in heart tissue sections from saline-(A-C) and rmAPN-treated groups(D-F) and overlay of signals showing the coexistence of both regions of the protein. Arrows: the typical tip to tip connections. Scale bar = 20 µm for all panels. G: Quantitative immunofluorescence analysis of N-cad signal in rmAPN- and saline-treated left ventricles. The quantification of signals is shown as the percentage of labelled area in the total heart tissue (*P < 0.05).

Western blot findings were also confirmed by the results of quantitative immunofluorescence (***Fig. 3***). The signal intensity of C-terminal of N-cad showed no significant difference between rmAPN-treated mice and saline-treated mice (0.052%±0.013% vs 0.051%±0.012%, *n* = 4, *P* > 0.05). However, the signal intensity of N-terminals of N-cad significantly decreased in rmAPN-treated mice compared to saline-treated mice (0.033%±0.007% *vs* 0.050%±0.010%, *n* = 4, *P* < 0.05). Coincident with the decreased N-terminal signals, the coexistence area of both terminals decreased significantly in rmAPN-treated mice (0.032%±0.007% *vs* 0.048%±0.008%, *n* = 4, *P* < 0.05).

### rmAPN affects both the distribution and phosphorylation of Cx43 *in vivo*

**Fig. 4:**
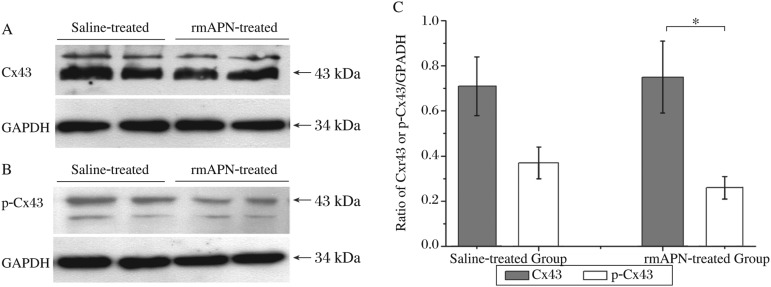
Effects of rmAPN on the phosphorylation of Cx43. A: Immunoblot of total Cx43 in heart tissues from both rmAPN- and saline-treated mice. B: Immunoblot of p-Cx43 in the heart tissue of both groups. C: The relative density of total Cx43 and p-Cx43 normalized to GAPDH (*P < 0.05).

Cx43 expression in whole-heart protein lysates was also probed by western blot. The relative density of total Cx43 signals was similar between the rmAPN-treated mice and the saline-treated mice (0.750±0.160 *vs* 0.710±0.130, *n* = 6, *P* > 0.05). However, the phosphorylation level of Cx43 decreased significantly in the rmAPN-treated mice compared to saline-treated mice (0.260±0.060 *vs* 0.370±0.070, *n* = 6, *P* < 0.05) (***Fig. 4***). The results indicated a decrease in phosphorylated Cx43 (p-Cx43) without the reduction of total Cx43 in heart tissue from rmAPN-treated mice.

**Fig. 5:**
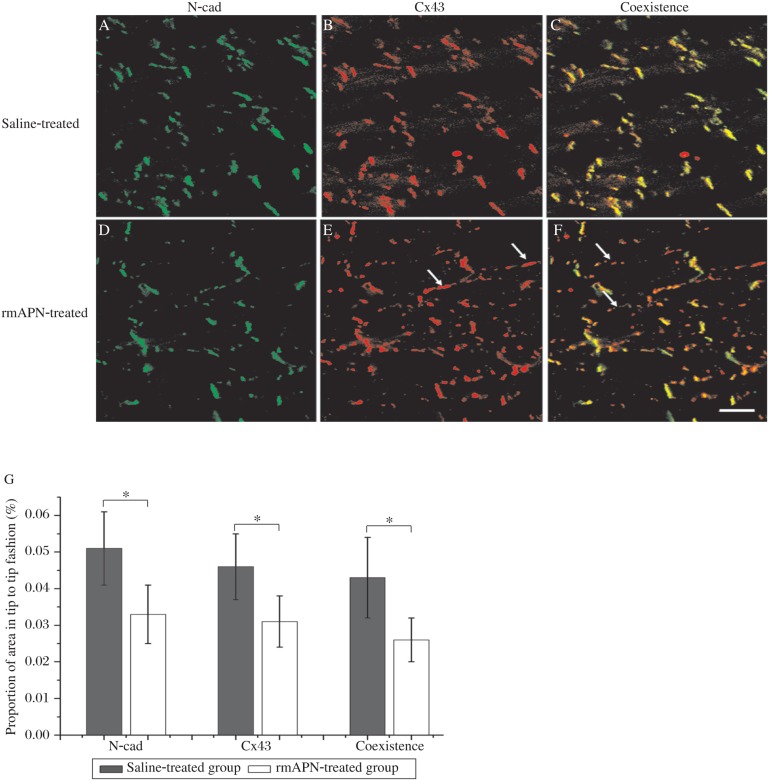
Redistribution of Cx43 in rmAPN-treated mice. A-F: Representative laser confocal images of left ventricular myocardia from rmAPN- and saline-treated mice immunostained with anti-Cx43 and anti-N-cad antibodies. A strong Cx43 signal (red) at the IDs throughout rmAPN-(D-F) and saline-treated heart tissues(A-C) and overlay of signals show the colocalization of the proteins. Arrows show the Cx43 signal redistribution onto the scarcolemma in rmAPN-treated hearts. Bar=20 µm for all the panels. G: Comparison of the signals of N-cad and Cx43 in tip-to-tip fashion in the heart tissue from both groups (*P < 0.05).

Heart tissue sections were double-labelled with anti-Cx43 and anti-N-cad (bound to the N-terminal) antibodies for confocal laser microscopy inspection. The specific high-intensity immunoreactive signals of Cx43 were identified at points of intercellular apposition (***Fig. 5A***). In rmAPN-treated mice, the amount of Cx43 signals from tip-to-tip contacts decreased significantly compared to the saline-treated group (0.031%±0.007% *vs* 0.046%±0.009%, *n* = 4, *P* < 0.05). However, the bilateral signals formed in the cells increased dramatically. The distribution of N-cad signals remained unchanged in both groups, but N-terminal signals of N-cad in the tip-to-tip fashion decreased significantly in rmAPN-treated mice compared to the saline-treated mice (0.033%±0.008% *vs* 0.051%±0.010%, *n* = 4, *P* < 0.05). The coexistence area of Cx43 and N-cad also decreased significantly in rmAPN-treated mice (0.026%±0.006% *vs* 0.043%±0.011%, *n* = 4, *P* < 0.05) in accordance with the decrease of Cx43 in the IDs (***Fig. 5B***). The above results show a significant decrease of Cx43 in IDs and redistribution on the bilateral sarcolemma.

### Susceptibility to ventricular arrhythmia increased in rmAPN-treated mice

Electrophysiological studies were performed on 20 rmAPN-treated and 22 saline-treated mice. The basic ECG features are shown in ***Table 1***. The heart rate (HR) of the rmAPN-treated mice was similar to the saline-treated animals. The HR, PR, RR and QT intervals were not significantly different. However, the QRS duration in rmAPN-treated mice was statistically longer than that in the saline-treated mice (14.70±1.25 ms *vs* 13.60±1.13 ms, *P* < 0.05).

**Table 1:** Electrocardiographic data and incidence of inducible ventricular arrhythmias

Group	Electrocardiographic data				
	HR(bpm)	PR interval (ms)	QT interval (ms)	RR interval (ms)	QRS duration (ms)
rmAPN-treated (*n* = 20)	437 ± 36	41.50 ± 3.60	41.20 ± 9.10	137.00 ± 13.10	14.70 ± 1.25
Saline-treated (*n* = 22)	454 ± 42	39.20 ± 2.80	40.30 ± 7.50	132.00 ± 9.20	13.60 ±1.13*

*P < 0.05 *vs* saline-treated group

**Fig. 6:**
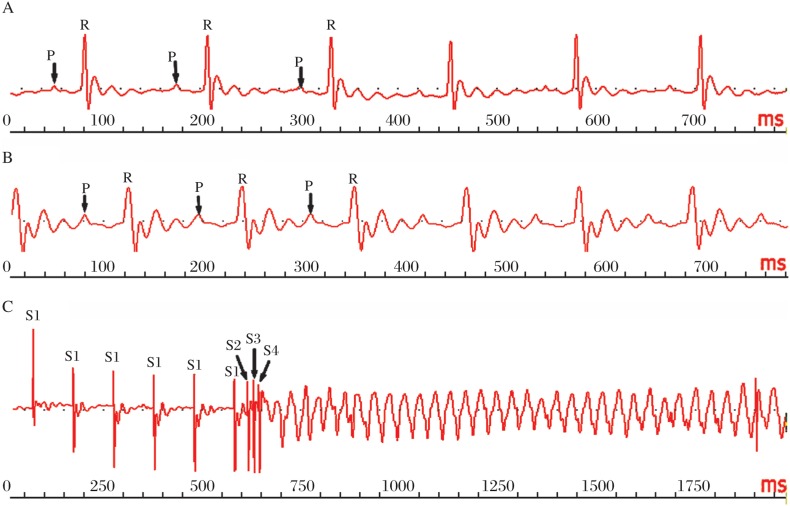
Electrocardiogram and electrophysiological studies on rmAPN-treated mice. A: Electrocardiogram at baseline (before infusion). B: Electrocardiogram after 60-min infusion of rmAPN. Surface ECG displayed widen QRS wave and delayed PR interval. C: Sustained ventricular tachycardia induced by programmed stimulation (S1/S2/S3/S4:100/60/40/20 ms).

Sustained ventricular tachycardia (VT) was observed in 10 rmAPN-treated mice and 1 saline-treated mouse when induced by PES (***Fig. 6***). Two rmAPN-treated mice suffered ventricular fibrillation(VF), but no VF was observed in the saline-treated group. Total incidence of inducible ventricular tachyarrhythmias was 50% (10/20) and 4.5% (1/22) in rmAPN-treated mice and saline-treated mice, respectively (*P* < 0.01). No spontaneous VT or VF occurred in either group.

## DISCUSSION

In our present study, we developed an rmAPN-treated mouse model to observe whether impaired cell-to-cell adhesion enhances the susceptibility to ventricular arrhythmia. The results showed that rmAPN caused extracellular N-cad N-terminal disruption, Cx43 redistribution and dephosphorylation in IDs between cardiomyocytes. Furthermore, the incidence of inducible ventricular tachyarrhythmias increased in the rmAPN-treated mice.

Previous studies showed that protease enzymes increased in the infarcted heart tissue and disrupted cell-to-cell and cell-extracellular matrix connections[18]–[20]. The loss of N-cad, consistent with a decrease of Cx43 at the border zone of infracted areas might result in impairment in cadherin-mediated adherence[21]. Alterations of Cx43 expression and remodeling of GJs at the border zone of the infarcted areas were known to cause slow and heterogeneous conduction[22]. However, the initiating mechanism of GJ remodeling by impaired adherence junctions in heart tissue has not been clarified. Lindsey *et al*[6] found defects in electrical conduction accompanied with decreased expression of Cx43 in the hearts of MMP-7-treated mice. They also showed evidence that MMP-7 directly bound to Cx43 and cleaved it. In our study, we demonstrated that rmAPN could rapidly disrupt adherence junctions *in vitro*, and *in vivo* infusion of rmAPN in mice could impair the adherence junctions by disrupting the N-terminal of N-cad. The total quantity of Cx43 did not decrease in the rmAPN-treated mice which was different from the decrease of Cx43 seen in the MMP-7-treated mice. The possible reason may be that rmAPN can't bind to the N-terminal of Cx43 due to the blockage of cytomembrane.

The reduction of Cx43 played the most important role in arrhythmogenesis[23]. van Rijen *et al*[24] demonstrated that blunted electric conduction occurred in induced Cx43-deletion mice, whose Cx43 was suppressed no less than 80%. However, Kostetskii *et al*[25] reported that only a 40% reduction of Cx43 was sufficient to cause ventricular arrhythmia and sudden death in N-cad conditional knockout mice. A loss of recognizable ID structures along with intercellular space separation between cardiomyocytes was also found. Therefore, we deduced that impaired adherence function might aggravate the blunted electrical conduction due to the loss of Cx43. Interestingly, the acute impairment in adherence junctions resulted in Cx43 redistribution onto the bilateral sarcolemma in the heart tissue, and caused a 35% reduction of Cx43 in the IDs of rmAPN-treated mouse hearts.

The phosphorylation/dephosphorylation of Cx43 regulates the internalization, degradation and channels gating properties[26],[27]. In acute ischemia, dephosphorylation of Cx43 plays an important role in the disorganization and electrical uncoupling of ventricular cells[28]–[30]. Our data provided evidence that compromised N-cadherin-mediated intercellular adhesion leaded to a significant dephosphorylation of Cx43. Therefore, our results implyed that impaired adhesion leaded to more dysfunctional GJs in the IDs between cardiomyocytes.

The changes of Cx43 may cause defects in the electrical conduction function of GJs and increase the risk of ventricular tachyarrhythmia. Our electrophysiological study showed a higher susceptibility to ventricular arrhythmias in the rmAPN-treated mice. Prolonged ventricular depolarization was associated with the dissolved N-cad in the rmAPN-treated mouse hearts was also observed in our study. The findings in our study indicate that cleavage of N-cad results in impairment of adherence junctions and increased susceptibility to ventricular arrhythmias in mice. The arrhythmogenic mechanism is probably, correlated with the loss of functional Cx43 in the IDs.

However, there are some limitations in our study. Firstly, because the rmAPN is a low-selectivity proteolytic enzyme, it possibly affected the function of other proteins, such as proteins involved in calcium homeostasis, which play a critical role in arrhythmias. Secondly, apoptosis, which is common in the arrhythmia-correlated conditions, has been considered as the mechanism associated with cardiac rhythm disorders for a long time[31],[32]. As an acute test, the effect of rmAPN on apoptosis was not involved in our study. Finally, interventions restricted to the adhesion function of N-cad are needed in subsequent investigations to determine the precise mechanism of impaired adherence junctions on the occurrence of ventricular arrhythmias.
